# Escalator: An Autonomous Scheduling Scheme for Convergecast in TSCH

**DOI:** 10.3390/s18041209

**Published:** 2018-04-16

**Authors:** Sukho Oh, DongYeop Hwang, Ki-Hyung Kim, Kangseok Kim

**Affiliations:** 1 Department of Computer Engineering, Graduate School of Ajou University, Suwon 16499, Korea; tadybear@ajou.ac.kr (S.O.); bc8c@naver.com (D.Y.H.); 2 Department of Cyber Security, Ajou University, Suwon 16499, Korea; kangskim@ajou.ac.kr

**Keywords:** wireless sensor network, TSCH, convergecast, autonomous scheduling, RPL

## Abstract

Time Slotted Channel Hopping (TSCH) is widely used in the industrial wireless sensor networks due to its high reliability and energy efficiency. Various timeslot and channel scheduling schemes have been proposed for achieving high reliability and energy efficiency for TSCH networks. Recently proposed autonomous scheduling schemes provide flexible timeslot scheduling based on the routing topology, but do not take into account the network traffic and packet forwarding delays. In this paper, we propose an autonomous scheduling scheme for convergecast in TSCH networks with RPL as a routing protocol, named Escalator. Escalator generates a consecutive timeslot schedule along the packet forwarding path to minimize the packet transmission delay. The schedule is generated autonomously by utilizing only the local routing topology information without any additional signaling with other nodes. The generated schedule is guaranteed to be conflict-free, in that all nodes in the network could transmit packets to the sink in every slotframe cycle. We implement Escalator and evaluate its performance with existing autonomous scheduling schemes through a testbed and simulation. Experimental results show that the proposed Escalator has lower end-to-end delay and higher packet delivery ratio compared to the existing schemes regardless of the network topology.

## 1. Introduction

Time Division Multiple Access (TDMA) and channel hopping-based MAC protocols are being developed for energy efficient and reliable communication in Wireless Sensor Networks (WSNs) composed of low-power wireless devices [1,2,3,4]. These protocols perform synchronous and deterministic communications based on timeslot schedules in units of the slotframe (or superframe), a group of timeslots that continuously repeats over time. TDMA uses a deterministic schedule to reduce idle listening and collisions in packet transmission. Channel hopping mitigates packet transmission failures that might occur due to multipath fading or channel conflicts through frequency diversity [5]. These protocols perform channel hopping in timeslots along the hopping pattern and provide simultaneous communications in a single timeslot using a channel offset. These protocols provide a low duty cycle and high reliability compared to existing asynchronous protocols [6].

In the WSNs based on TDMA and channel hopping, routing and timeslot scheduling schemes are important because they determine the performance of the entire network. Routing determines the packet forwarding path of the network. Reliable link-based robust routing protocols provide reliability for the end-to-end packet delivery. The timeslot schedule of the TDMA network determines the end-to-end delay of the packet transmission and the bandwidth of the network. The timeslot schedules that generate consecutive timeslots along the packet forwarding path minimize the end-to-end delay of the packet transmission. Furthermore, the timeslot schedules that minimize the slotframe size (or length) increase the bandwidth of the network.

Several scheduling schemes have been proposed for WSNs based on TDMA and channel hopping. Scheduling schemes could be classified by the entities that create and manage the schedules [7,8]. Firstly, in centralized scheduling, a central entity performs the creation and management of the schedules. The central entity collects properties such as traffic requirements, network topology information and link status from the nodes to create schedules. Secondly, in the distributed scheduling, each node in the network performs the creation and management of the schedules through schedule information exchange and negotiations with neighbor nodes. Finally, in the autonomous scheduling, there is no entity that performs the creation and management of the schedules [8]. Autonomous scheduling determines the schedule according to the pre-established rules without information collections or negotiations among nodes for schedule creation and management, thereby reducing the collection overhead.

One of the common communication patterns in WSNs is convergecast, which means the data collection from all nodes to the sink opposite to broadcast. In general, convergecast means that all nodes in the network transmit data generated to the sink periodically. Event-driven data collection is also a convergecast in a broad sense [9]. Because the data collection is one of the most typical WSN applications, the convergecast could be considered as the main communication pattern of WSNs. At the convergecast, all nodes must be able to forward packets to the sink until the next packet generation cycle.

In this paper, we propose an autonomous scheduling scheme, named Escalator, for the convergecast in a TSCH network with the RPL [10] routing protocol. The main advantage of Escalator is that it creates a conflict-free schedule, which minimizes the end-to-end delay, without additional information exchange for scheduling. In Escalator, each individual node generates and manages a timeslot schedule in an autonomous manner for a convergecast based on the routing topology generated by RPL. That is, each node manages a timeslot schedule as soon as the node receives the RPL control messages such as DIO (Destination-Oriented Directed Acyclic Graph (DODAG) Information Object) or DAO (Destination Advertisement Object) when the network topology changes. Escalator generates a conflict-free schedule that allows all nodes in the network to send packets to the sink in every slotframe cycle. Escalator minimizes the end-to-end delay by creating a consecutive timeslot schedule along the packet forwarding path from a node to the sink.

We implement Escalator and evaluate the performance with the existing autonomous scheduling schemes in a testbed. We also perform an extended evaluation in various aspects through simulations. In the evaluation results, Escalator shows better performance in the packet delivery ratio and the end-to-end delay than the existing autonomous schemes. In terms of the energy efficiency, the duty cycle of a node is more susceptible to the size of the routing table entries compared to the existing schemes. In other words, the duty cycle of the node in Escalator is generally similar to that of the existing schemes, but the duty cycle becomes higher as the routing table entry increases.

The main contributions of this paper are as follows:

1. We propose Escalator, a new autonomous timeslot and channel scheduling scheme for the convergecast in TSCH-based WSNs with RPL.
2. We prove that Escalator generates a conflict-free schedule.
3. We show through experiments that Escalator improves the packet delivery ratio, the end-to-end delay and the average energy efficiency for the convergecast compared to the existing autonomous schemes.

The rest of this paper is organized as follows. In Section 2, we introduce TSCH and RPL, which form the basis of this study. In Section 3, the existing timeslot scheduling algorithms are discussed. Section 4 proposes the overall operation of Escalator. In Section 5, we prove the conflict-freeness of the generated schedules and analyze the packet transmission delay and bandwidth. In Section 6, we evaluate the performance of Escalator using a testbed and simulations compared to the existing autonomous scheduling schemes. Finally, the paper is concluded with future research directions in Section 7.

## 2. Background

This section introduces TSCH and RPL, which are the basis for Escalator.

### 2.1. TSCH Overview

Time Slotted Channel Hopping (TSCH) is the MAC protocol defined in IEEE 802.15.4e-2012 [4]. The TSCH uses a slotframe to provide synchronized communications to the network. The slotframe is a collection of timeslots that are repeated over time. The timeslot is the basic unit of communication in TSCH, defined as a time sufficient for a pair of devices to exchange a frame and an acknowledgment. In each timeslot, nodes could perform one of the following operations: transmit, receive and idle. The communication between the nodes is established by the timeslot schedule, and the size of the slotframe determines how often the timeslot schedule repeats. When a node transmits a packet over a channel in a timeslot, one or more other nodes are scheduled to receive it over the same channel in the timeslot.

The TSCH provides channel hopping so that the node can change the communication channel for each timeslot. The channel hopping of the TSCH removes collisions due to wireless interference and provides frequency diversity to mitigate multipath fading. A node in the TSCH network selects a channel to use for communication along a predefined hopping pattern. In channel hopping, a channel offset is used to change the communication channel. Two or more links occurring in the same timeslot can communicate without overlap using different channel offsets.

TSCH provides a time synchronization mechanism using a beacon. The TSCH beacon consists of the information required for the network joining including the network time. Nodes in the TSCH network periodically advertise the beacon to one another. A TSCH node synchronizes its clock time with other TSCH nodes via the advertised beacon.

Internet Engineering Task Force (IETF) standardizes 6TiSCH (IPv6 over the TSCH mode of IEEE 802.15.4e) [11] for IPv6 communication based on TSCH. 6TiSCH combines TSCH with the existing well-defined IPv6 protocol stack for WSNs. 6TiSCH supports both centralized scheduling such as the Path Computation Element (PCE) [12] and decentralized scheduling based on the 6top Protocol (6P) [13] for scheduling of the MAC layer not covered by the TSCH standard. 6TiSCH uses RPL [10] as a routing protocol.

### 2.2. RPL

The Routing Protocol for Low-power and lossy networks (RPL) [10] is a gradient-based distance vector routing protocol for WSNs supporting diverse link layers. RPL uses a Destination-Oriented Directed Acyclic Graph (DODAG) as a routing topology. The DODAG is a directional graph in which all edges are directed at the root node. An RPL node could transmit packets towards the root node using the DODAG topology. In RPL, nodes generate and manage routing information using the RPL control messages such as the DODAG Information Object (DIO) and Destination Advertisement Object (DAO). The node in the DODAG periodically advertises the DIO messages, which include configuration attributes that all RPL nodes should have. The RPL node receiving the DIO message advertised by the other node participates in the DODAG. At this time, the RPL node selects the source node of the DIO message as the parent. When an RPL node participates in a DODAG, it transmits the DAO messages to its parent. The RPL node receiving a DAO message generates the downward routing information for the source node of the DAO message and then transmits the message to the parent. This DAO message transfer is repeated until the DAO message reaches the root node, which creates a downward path from the root node to the source node of the DAO message.

## 3. Related Work

### 3.1. Scheduling of WSNs based on TDMA and Channel Hopping

TDM-A and channel hopping-based WSNs require scheduling to generate timeslot and channel schedules for communication between nodes. The number and order of timeslots scheduled for a node determine the throughput of the node and the end-to-end delay of the packet transmission. In addition, the scheduling of timeslots and channels considering both the interference of wireless networks and the conflicts of the schedule can improve the reliability of the network. Since scheduling determines the performance of the network, an efficient scheduling scheme is required to improve the network performance. Several schemes have been proposed for efficient scheduling. These schemes could be classified into centralized, decentralized and autonomous scheduling schemes, depending on which the scheduling is actually performed.

The centralized scheduling schemes aim at generating an optimal schedule by using the information such as the link status and traffic requirements of the nodes collected by the central entity of the network such as the system manager. The Industrial Wireless Sensor Networks (IWSNs) such as Wireless HART and International Society of Automation (ISA) 100.11a, use the centralized scheduling for high reliability and real-time performance. Ergen et al. [14] proved that the scheduling problem is NP-complete and suggested both the node-based and the level-based heuristic scheduling algorithms. Han et al. [15] proposed a reliable path generation algorithm based on Wireless HART. The authors define reliability requirements of the graph and propose algorithms for graph generation and timeslot assignment satisfying the requirements. In TASA (Traffic Aware Scheduling Algorithm) [16], the central entity generates a schedule based on the network connectivity graph and traffic requirements, taking into account the conflicts and interactions between links.

In the distributed scheduling schemes, unlike the centralized schemes, scheduling functions are distributed across nodes. The nodes exchange information such as the link status and traffic requirements and use this information to determine the schedule. Therefore, the distributed scheduling can respond to network changes more quickly than the centralized schemes, which collect information from the central entity and determine the schedule. Tinka et al. [17] proposes a decentralized scheduling algorithm for mobile nodes in the TSCH network. Mobile nodes maintain their network connectivity through advertisements and link creations using a rendezvous slot. DeTAS (Decentralized Traffic Aware Scheduling) [18] generates a macro-schedule that assigns non-overlapping timeslot sections per DODAG in a multi-sink topology. In addition, each DODAG root performs a micro-schedule with respect to the traffic of the nodes for the assigned timeslot section. Domingo-Prieto et al. [19] proposed a distributed scheduling scheme based on a Proportional, Integral and Derivative (PID) control algorithm. Nodes calculate the required number of timeslots using the number of packets in the queue at the start of the slotframe and perform the negotiation and reservation of timeslots with neighbor nodes using the 6top protocol [13].

DeAMON (Decentralized Adaptive Multi-hop scheduling for 6TiSCH Networks) [20] gradually creates a schedule in the direction toward the root node starting from a leaf node that has no children. Through this, sequential schedules considering the packet forwarding path are generated to minimize the end-to-end latency. Furthermore, each node collects the schedule information of neighboring nodes through overhearing and generates a schedule capable of parallel communication using the schedule information. The Leapfrog Collaboration [21] provided path diversity using redundant paths. The nodes select an alternative parent in addition to the default parent and create a timeslot schedule so that the same packet can be sent to both parents. Packets received in duplicate during packet transmission are discarded. These multipath packet transmissions may improve the delay and reliability of the packet transmission. Multihop and Blacklist-based Optimized TSCH protocol (MABO-TSCH) [22] proposes a link-specific channel offset allocation scheme using graph coloring and an improved channel blacklisting scheme. Blacklist information of the communication channel is generated at one node of each parent/child pair, and this information is embedded in data or ACK frames exchanged between the two nodes. Hosni et al. [23] proposed a distributed scheduling based on the spectrum, which means the node group of the same hop count from the root. This scheme allocates a time-frequency block for each spectrum for collision avoidance. The time-frequency blocks are arranged in a sequential manner to reduce the packet transmission delays. These studies propose efficient distributed scheduling schemes that reduce delays through sequential schedules and parallel transmission. However, these distributed scheduling schemes use signaling or overhearing for information gathering from neighboring nodes. Our proposed scheme generates sequential schedules that support parallel transmission, such as the distributed scheduling schemes, but uses only routing information without additional overheads for scheduling. This is the difference between the existing distributed scheduling schemes and Escalator.

The state of the art solution is autonomous scheduling. In autonomous scheduling, each node in the network determines the communication schedule like distributed scheduling. However, unlike distributed scheduling, each node schedules according to predefined rules without any signaling and resources to the schedule creation or modification. The autonomous scheduling has the advantage of less overheads for scheduling than the centralized and distributed schemes. However, there is a disadvantage that it is difficult to generate an optimal schedule. Orchestra [8] is an autonomous scheduling scheme based on the routing information. Orchestra uses multiple slotframes separated by traffic types. Orchestra uses the node ID and routing information for the three slotframes to generate a schedule that considers the routing topology. However, this schedule is not suitable for data collection, which is a typical application of WSNs because the delay and traffic requirements are not considered. 6TiSCH-minimal [24] can be seen as an autonomous scheduling scheme in a large category because it does not use a separate scheduler. 6TiSCH-minimal provides a schedule with a single shared timeslot in that all nodes in the network use it in a slotted-aloha fashion. 6TiSCH minimal is used as the baseline schedule of 6TiSCH and can be used with other scheduling schemes.

### 3.2. Timeslot Scheduling for Convergecast

The convergecast is a common communication pattern in WSNs and implies the data collection from many or all nodes to a sink. There are many studies to minimize both the delay from nodes to the sink and the total convergecast time in order to improve the convergecast performance of the TDMA network. Gandham et al. [25] introduced a distributed scheduling algorithm for the convergecast in a single channel TDMA-based WSN. The algorithm uses a slotframe of size N×3 in a general network with *N* nodes. Incel et al. [26] proved that the conflict-free scheduling problem using the least number of channels in the multichannel TDMA WSNs is NP-complete. Zhang et al. [27] proposed a link scheduling and channel assignment algorithm for the convergecast in a linear topology WirelessHART network. The authors propose a polynomial time scheduling algorithm using a slotframe with the size N×2−1 and the number of channels ⌈N/2⌉ for a linear topology network consisting of *N* single buffer nodes. Soua et al. [28] proposed a distributed scheduling algorithm aimed at minimizing the slotframe size required for the convergecast. This algorithm divides the slotframe with a unit called the wave and generates waves repeatedly to form a schedule for all nodes in the network to transmit packets generated at regular intervals to the sink.

We propose an autonomous scheduling scheme for the convergecast, which is different from the previous studies. Our proposed scheme automatically generates a schedule considering both the node’s traffic requirements and the delay of packet transmissions. In the next section, we describe the proposed scheme in detail.

## 4. Proposed Autonomous Scheduling Scheme for Convergecast

In this paper, we propose Escalator, an autonomous scheduling scheme for convergecast in TSCH-based WSNs with RPL. The basic idea of Escalator is to create a pipeline-like communication schedule. Each node allocates two timeslots for each descendant node included in its sub-graph. In one timeslot, a node receives a packet generated from a descendant node, and in the next timeslot, the node sends the received packet to its parent immediately. In addition, Escalator adjusts the schedule and determines the communication channel so that the packet can be continuously transmitted in chronological order from the source to the sink using the allocated timeslots.

Escalator, operating on each node of the network, generates and manages timeslot and channel schedules for convergecast using only local routing information of the node without signaling or negotiation with other nodes. The generated schedules of Escalator on every nodes have two main characteristics: Firstly, it is conflict-free. Secondly, it is a consecutive timeslot schedule along the packet forwarding path, thereby reducing the end-to-end delay.

Escalator uses routing topology information and hop counts for scheduling and operation. The hop count of a node means the hop distance to the DODAG root, and we assume that the node may know the hop count by the routing protocol. Since Escalator uses only the generated routing topology, the routing topology generation method does not affect the operation of Escalator. In other words, Escalator can be applied regardless of the Object Function (OF) of RPL.

**Figure 1:**
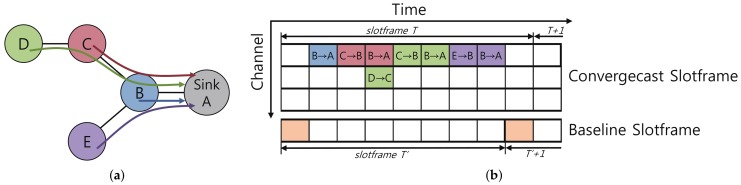
An example of the Escalator schedule: (**a**) network topology of the example; (**b**) Escalator’s generated schedule for the example.

In Escalator, there are two types of slotframes with their own sizes: convergecast and baseline slotframes. The convergecast slotframe is used for carrying both TSCH beacons and the convergecast data of nodes, while the baseline slotframe is for RPL route control messages and downward data traffic (i.e., packets sent from the sink to nodes). Since RPL control messages are used to create and update the routing topology in real time, Escalator gives the baseline slotframe a higher priority than the convergecast slotframe. Figure 1 shows an example of a schedule generated by Escalator. Each timeslot in Figure 1b shows a transmission of data, and its background color matches with the color of the originating node of the transmitted data in Figure 1a. Convergecast and baseline slotframes are also shown in Figure 1b, separately. The size of the two slotframes are not necessarily the same. The generated schedules in the convergecast slotframe of Figure 1b are consecutive so that the data could be delivered to the sink from any node in a single convergecast slotframe.

To explain the operation of Escalator, we define a network as follows. The network G=(V,E) consists of *N* nodes, and all of the nodes $v_{i}\in V$ have unique ID {i|1≤i≤N}. The ID of the node is pre-installed, and we assume that the node ID is managed by a central entity such as the DODAG root. Assigning a preconfigured unique ID to a node can be sufficiently assumed in a managed network, so it would not be a challenge in practical applications and implementations. All nodes in the network participate in DODAG using RPL, and the DODAG root acts as a sink. Each node $v_{i}$ has its preferred parent $p(v_{i})$, its direct child set $C(v_{i})$ and its sub-graph’s nodes set $SG(v_{i})$. For nodes $v_{i},v_{j}\in V$, $l_{ij}\in E$ is defined as a link between the sender $v_{i}$ and the receiver $v_{j}$. $H_{v_{i}}$ means the number of hops from the sink to node $v_{i}$. The network performs channel hopping using Ch orthogonal channels. Data are generated from all nodes except the sink and are transmitted to the sink according to schedules specified for each node. Table 1 summarizes the main notations in this paper.

The operation of Escalator consists of four parts: timeslot allocation, sliding slotframe, channel offset selection and baseline slotframe size determination. Firstly, the timeslot allocation mechanism allocates two dedicated timeslots to each node for the receive and transmit operations, respectively. Secondly, the sliding slotframe mechanism shifts its allocated timeslots for pipelined transmission between parent and child nodes. Thirdly, the channel offset selection mechanism minimizes timeslot usage by parallelizing concurrent transmissions of packets. Finally, the baseline slotframe size determination mechanism finds the optimal baseline slotframe size for minimizing the conflicts between the convergecast and baseline slotframes. The detailed operation of each mechanism is described in the next section.

### 4.1. Timeslot Allocation Mechanism

The timeslot allocation mechanism of Escalator in a node generates a timeslot schedule for the convergecast slotframe that uses only the node ID and routing topology information without additional signaling with other nodes. The generated Escalator schedule of node $v_{i}$ consists of the timeslots with the following four operations for the TSCH beacon and convergecast traffic transmission.

- $BT_{i}$: The operation in which $v_{i}$ broadcasts a beacon
- $BR_{ij}$: The operation in which $v_{i}$ receives a beacon sent by $v_{j}$
- $TX_{ij}$: The operation in which $v_{i}$ transmits a unicast packet originated from $v_{j}$ to the parent node $p(v_{i})$
- $RX_{ij}$: The operation in which $v_{i}$ receives a unicast packet, originated from $v_{j}$, from one of the child nodes $C(v_{i})$

The timeslot allocation mechanism consists of the following five allocation rules that set operations to timeslots.

- SET_BT(i): Node $v_{i}$ sets $BT_{i}$ to i×2−1 timeslot (for example, $BT_{1}$ = 1, $BT_{2}$ = 3, $BT_{3}$ = 5, $BT_{4}$ = 7).
- SET_BR(i,j): Node $v_{i}$, where $v_{i}\neq sink$, sets $BR_{ij}$ to j×2 timeslot to receive a broadcast beacon from $v_{j}=p\left(v_{i}\right)$ (for example, $BR_{21}$ = 2, $BR_{32}$ = 4, $BR_{42}$ = 4).
- SET_TX(i,i): Node $v_{i}$, where $v_{i}\neq sink$), sets $TX_{ii}$ to i×2 timeslot to send its unicast packet to $v_{i}$’s parent $v_{j}=p\left(v_{i}\right)$ (for example, $TX_{22}=4$, $TX_{33}=6$, $TX_{44}=8$).
- SET_RX(i,j): Node $v_{i}$ sets $RX_{ij}$ to the j×2−1 timeslot to receive the unicast packet generated by the node $v_{j}\in SG\left(v_{i}\right)$ (for example, $RX_{12}=3$, $RX_{13}=5$, $RX_{14}=7$, $RX_{23}=5$, $RX_{24}=7$).
- SET_TX(i,j): Node $v_{i}$, where $v_{i}\neq sink$, sets $TX_{ij}$ to j×2 timeslot to transmit the packet generated by $v_{j}\in SG\left(v_{i}\right)$ to $v_{k}=p\left(v_{i}\right)$, the parent of vi(for example, $TX_{23}=6$, $TX_{24}=8$).

**Figure 2:**
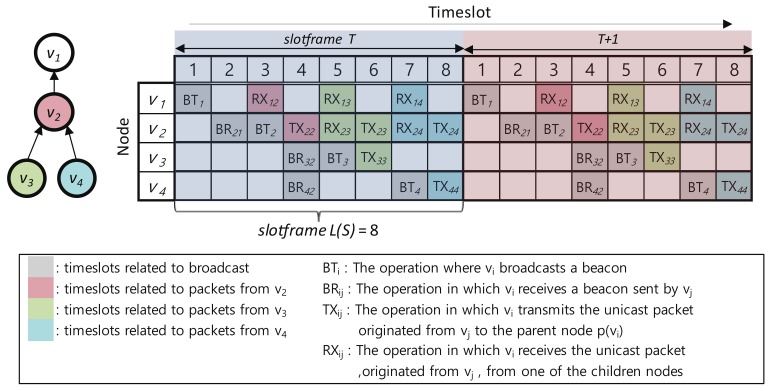
An example of timeslot allocation for the convergecast slotframe.

Figure 2 shows a schedule with a convergecast slotframe size $L(S_{Conv})=8$ in a network consisting of four nodes with sink $v_{1}$. In Figure 2, the gray timeslots are used to broadcast the beacon, and the red, green and blue timeslots are used to deliver packets generated by $v_{2}$, $v_{3}$ and $v_{4}$, respectively.

The timeslot allocation mechanism uses the above-mentioned allocation rules when a node receives RPL control messages to reflect changes in the DODAG topology into the node’s timeslot schedule. Algorithm 1 shows the application of the allocation rules when a node receives RPL control messages. When a node participates in a DODAG through the reception of a DIO, the node adds TX, BT (Broadcast Transmission) and BR (Broadcast Reception) operations for transmitting the node’s own packets and beacons to the convergecast slotframe. If a node already participating in the DODAG receives a DIO, the node checks whether the routing topology has changed. If the routing topology has changed, the node removes the BR operation that was used in the previous topology and adds a new BR operation for the new topology to the convergecast slotframe. When a node receives a DAO, the node adds TX and RX operations to the convergecast slotframe for forwarding packets originating from a node that sent the DAO. In Figure 2, node $v_{4}$ sends a DAO to $v_{2}$. When node $v_{2}$ receives the DAO of node $v_{4}$, node $v_{2}$ allocates $RX_{24}$ and $TX_{24}$ to Timeslots 7 and 8, respectively, and delivers the DAO to node $v_{1}$. Node $v_{1}$ that has received the DAO of node $v_{4}$ from node $v_{2}$ allocates $RX_{14}$ to Timeslot 7.

**Algorithm 1** RPL callback handler for timeslot allocation. 1: **procedure**
RPL callback handler(*cm*, *j*)▹*j* is a sender of the RPL control message *cm* 2:  i← ID of $v_{i}$▹ set *i* to node’s own ID 3:  k← ID of p(i)▹ get parent ID of node $v_{i}$ 4:  s← ID of sink▹ get ID of sink 5:  **if**
cm = DIO **then** 6:   **if** route updated **then** 7:    **if** first joining of DODAG **then** 8:     **if**
i≠s
**then** 9:      SET_TX(*i*, *i*)10:      SET_BR(*i*, *j*)11:     **end if**12:     SET_BT(*i*)13:    **else if**
*j*≠*k*
**then**14:     UNSET_BR(*i*, *k*)15:     SET_BR(*i*, *j*)16:    **end if**17:   **end if**18:  **else if**
cm = DAO **then**19:   SET_RX(*i*, *j*)20:   **if**
i≠s
**then**21:    SET_TX(*i*, *j*)22:   **end if**23:  **end if**24: **end procedure**

### 4.2. Sliding Slotframe Mechanism

The sliding slotframe mechanism, which is responsible for running schedules in Escalator, forms a communication link between the parent and child nodes using the timeslot schedule assigned to the convergecast slotframe. In the TSCH network, a node forms a communication link with another node using the current slot number calculation. However, the timeslot schedule of the convergecast slotframe generated by the timeslot allocation mechanism in Section 4.1 could not form links using the existing slot number calculation. Through the new slot number calculation provided by the sliding slotframe mechanism, the operation assigned to the timeslot of one node’s convergecast slotframe could form a link with the operation of the other node.

The TSCH node determines the operation of the current timeslot by calculating the current slot number for each slotframe using the Absolute Slot Number (ASN). ASN is the total number of timeslots that have elapsed since the start of the network. The current slot number ${ts}_{S}$ at the slotframe *S* could be obtained with Equation (1) in TSCH [4].

(1) $${ts}_{S}=ASN\;\mathrm{mod}\;L(S),$$

where L(S) is the size of slotframe *S*.

The current slot number calculation of Escalator adds the hop count of the node to Equation (1). The current slot number, $ts_{S_{Conv}}\left(v_{i}\right)$, at the convergecast slotframe $S_{Conv}$ at a node $v_{i}$ could be obtained as Equation (2).

(2) $$ts_{S_{Conv}}\left(v_{i}\right)=\left(ASN+H_{v_{i}}\right)\;\mathrm{mod}\;L\left(S_{Conv}\right),$$

where $S_{Conv}$ is the convergecast slotframe, $L(S_{Conv})$ is the size of $S_{Conv}$ and $H_{v_{i}}$ is the hop count of $v_{i}$.That is, the timeslot schedule of the convergecast slotframe for a node with the sliding slotframe mechanism is shown as being left shifted by the hop count of the node.

**Figure 3:**
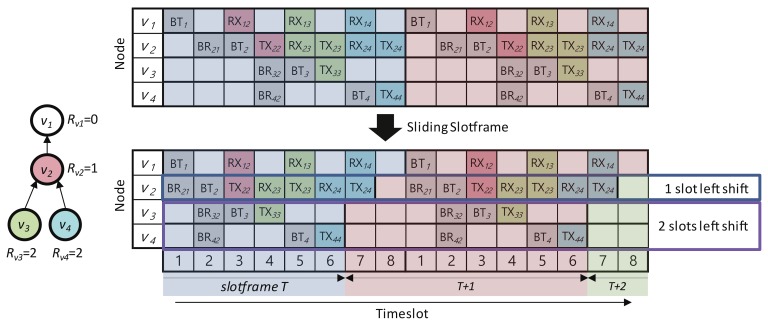
An example of the sliding slotframe.

Figure 3 shows an example of applying the sliding slotframe mechanism to the schedule in Figure 2. The schedule for node $v_{2}$ is left shifted by $H_{v_{2}}=1$. The schedules for node $v_{3}$ and $v_{4}$ are left shifted by $H_{v_{3}}=H_{v_{4}}=2$. These schedule shifts allow the TX and RX operations of the nodes in the example to be paired with each other, and communication links are formed between them.

### 4.3. Channel Offset Selection Mechanism

**Figure 4:**
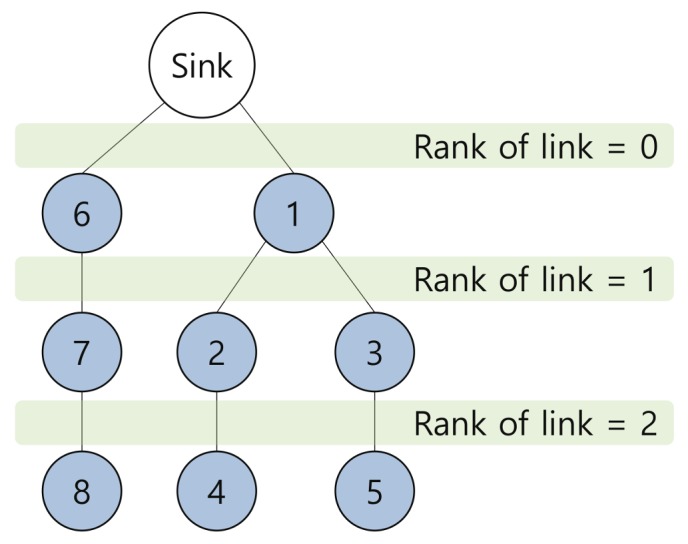
An example of the rank of links.

This section describes the channel offset selection mechanism that determines the concurrent communication channel usage in Escalator. That is, the selection mechanism assigns different channels to the communication links that could be in conflict with each other for concurrent communication by utilizing the rank of the links. The rank of a link means the distance from the sink to the link and is determined by the smaller value between the hop counts of the two nodes constituting the link. Figure 4 shows the ranks of links. The channel offset selection mechanism allocates a channel offset for the links with two adjacent ranks (for example, links with Ranks 0 and 1 use Channel Offset 0, and links with Ranks 2 and 3 use Channel Offset 1). Due to the nature of this channel allocation, Escalator has the following network extension range: When the number of available channels in the network is Ch, the maximum hop count of nodes in the network is less than or equal to Ch×2.

The channel offset selection mechanism determines the channel offset by the rank of the link and the link communication type. There are two different link communication types in Escalator: unicast and broadcast. The unicast link of a node is used to receive packets from the child nodes and to transmit packets to the parent node, and the broadcast link of a node is used to advertise beacons to the child nodes. The direction of unicast and broadcast links in the same rank is opposite. Notice that the convergecast slotframe of Escalator deals with only upward unicast links and downward broadcast links because the downward unicast links are covered in the baseline slotframe. We define the channel offset calculation formulas for each link type so that links of the same rank use the same channel offset regardless of the type and direction of the link. Equations (3) and (4) are used to calculate the channel offset of the broadcast link of node $v_{i}$. Equations (5) and (6) are used to calculate the channel offset of the unicast link of node $v_{i}$.

(3) $$\begin{array}{c} \begin{array}{cc} {CO}_{BT_{i}} & =\left\lfloor \frac{H_{v_{i}}}{2}\right\rfloor \end{array} \end{array}$$

(4) $$\begin{array}{c} \begin{array}{cc} {CO}_{BR_{ij}} & =\left\lfloor \frac{\left(H_{v_{i}}-1\right)}{2}\right\rfloor ,\;\forall v_{j}\in V \end{array} \end{array}$$

(5) $$\begin{array}{c} \begin{array}{cc} {CO}_{TX_{ij}} & =\left\lfloor \frac{\left(H_{v_{i}}-1\right)}{2}\right\rfloor ,\;\forall v_{j}\in V \end{array} \end{array}$$

(6) $$\begin{array}{c} \begin{array}{cc} {CO}_{RX_{ij}} & =\left\lfloor \frac{H_{v_{i}}}{2}\right\rfloor ,\;\forall v_{j}\in V \end{array} \end{array}$$

Algorithm 2 shows the pseudocode of the function that determines the operation and channel offset at the current timeslot, which is executed on every node at every timeslot. This function uses ASN to calculate the timeslot of both convergecast and baseline slotframes (see Equations (1), (2) and Lines 2, 11 of the algorithm) and selects the operation accordingly. This function then calculates the channel offset to use for the selected operation (see Equations (3)–(6) and Lines 5–8 of the algorithm). That is, when a node executes the operation of the convergecast slotframe, it uses the channel offset selection mechanism to determine the channel offset according to the type of links. When the node executes the operation of the baseline slotframe, the channel offset is set to zero.

**Algorithm 2** Decide current timeslot operation and channel offset selection of the convergecast slotframe. 1: **procedure**
Get current operation and channel offset(ASN, *i*) 2:  $ts\leftarrow \left(ASN+H_{v_{i}}\right)\;\mathrm{mod}\;L\left(S_{Conv}\right)$▹ relative timeslot number in current $S_{Conv}$ 3:  $op\leftarrow OP(S_{Conv},ts)$▹ get operation for ts from $S_{Conv}$ 4:  **if**
op≠idle
**then** 5:   **if**
op=TX
**then**
$CO\leftarrow (H_{v_{i}}-1)/2$ 6:   **else if**
op=RX
**then**
$CO\leftarrow H_{v_{i}}/2$ 7:   **else if**
op=BT
**then**
$CO\leftarrow (H_{v_{i}}-1)/2$ 8:   **else if**
op=BR
**then**
$CO\leftarrow H_{v_{i}}/2$ 9:   **end if**10:  **end if**11:  $ts^{\prime }\leftarrow \left(ASN\right)\;\mathrm{mod}\;L\left(S_{Base}\right)$▹ relative timeslot number in current $S_{Base}$12:  $op^{\prime }\leftarrow OP\left(S_{Base},ts^{\prime }\right)$▹ get operation for $ts^{\prime }$ from $S_{Base}$13:  **if**
$op^{\prime }\neq idle$
**then**▹ if the operation of $S_{Base}$ is not idle14:   $op\leftarrow op^{\prime }$▹ the operation of $S_{Conv}$ is suppressed15:   CO←016:  **end if**17:  **return**
op,CO18: **end procedure**

**Figure 5:**
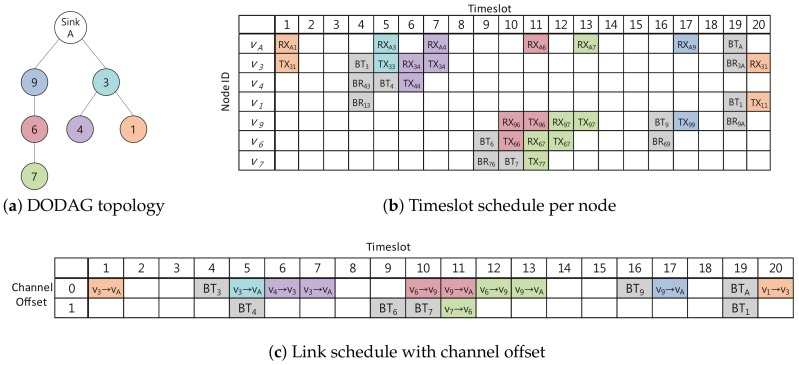
An example of the Escalator schedule. DODAG, Destination-Oriented Directed Acyclic Graph.

Figure 5 shows an example of a schedule for the convergecast slotframe. For a network consisting of seven nodes as shown in Figure 5a, Figure 5b is a schedule for each node of the convergecast slotframe with $L(S_{Conv})=20$ (i.e., N=10). Figure 5c shows a schedule of the convergecast slotframe with the selected channel offsets. In Figure 5c, links with Ranks 0 and 1 use Channel Offset 0, while links with Rank 2 use Channel Offset 1. Notice that we can see at Timeslots 5, 10, 11 and 19 that two or more communications in one timeslot are executed concurrently using different channel offsets.

The convergecast slotframe provides a schedule for delivering packets generated from all nodes in the network to the sink with minimal delay. Using the schedule shown in Figure 5c, a node can transmit packets to the sink using the same number of consecutive timeslots as the hop count of the node. The packet at node $v_{7}$ is delivered to the sink using three consecutive timeslots (green cells at Timeslots 11, 12 and 13 of Figure 5c). For packet delivery to the sink, nodes $v_{3}$ and $v_{9}$ use only one timeslot based on their hop counts as shown in Timeslots 5 and 17 of Figure 5c, respectively. Nodes $v_{1}$, $v_{4}$ and $v_{6}$ each use two consecutive timeslots based on their hop counts. Our proposed channel scheduling scheme was named Escalator because the packets generated in the nodes are sent up the stairs along the autonomously-generated consecutive timeslot based on the network topology.

### 4.4. Baseline Slotframe Size Determination

In Escalator, the baseline slotframe is used to transmit routing messages and downward traffic. The baseline slotframe $S_{Base}$ of length $L(S_{Base})$ consists of a single shared timeslot for all traffic and idle timeslots. All nodes in the network use this shared timeslot to create the DODAG topology with RPL. We give the baseline slotframe a higher priority than the convergecast slotframe, because the schedule of the convergecast slotframe depends on the DODAG topology information, which is generated from the baseline slotframe.

The baseline and convergecast slotframes are executed simultaneously in Escalator, and thus, a collision may occur between the two slotframes’ schedules. The collision between the two schedules means that the number of operations that a node must perform in one timeslot is two or more. If a collision occurs between the two simultaneous schedules of the two slotframes at any timeslot, the operation of the convergecast slotframe is suppressed, because its priority is lower than that of the baseline slotframe. The transmission of the convergecast traffic scheduled in the timeslot where the collision occurred is delayed until the next cycle of the convergecast slotframe. If a collision occurs at the same timeslot in the next cycle again, the convergecast traffic scheduled in the timeslot is delayed again. In order to deliver data packets to the sink with minimal delay, this schedule delay from the schedule collisions should be bound to the minimum. We set the size of the baseline slotframe so that convergecast traffic can be transmitted with minimal delay even if there is a collision between schedules. If the size of the baseline slotframe is the same as the convergecast slotframe, the operation using a particular timeslot of the convergecast slotframe might be continuously suppressed due to collisions at the specific timeslot of the baseline slotframe. Then, links that use the timeslot will not be able to send traffic. To solve this problem, we define conditions for the size of the baseline slotframe to prevent the continuous suppression of certain timeslots.

Before describing the conditions for the size of the baseline slotframe, we define the maximum hop counts of the packet forwarding path $H_{max}\left(G\right)$ of the network *G* in Escalator as follows:

(7) $$H_{max}\left(G\right)\leq \min\left(N-1,\;Ch\times 2\right),$$

where *N* is the number of nodes in the network and Ch is the number of available channels. For graph $G_{l}$ in a linear topology consisting of *N* nodes, the hop counts of the packet forwarding path from a node to the sink can be as long as N−1 hops. Escalator allocates one channel per two hops using the channel offset selection mechanism of Section 4.3, so the hop counts of the packet forwarding path for the number of available channels Ch can be as long as Ch×2 hops. In summary, the maximum hop counts of the packet forwarding path for graph *G* is less than or equal to the smaller value of the two cases.

**Figure 6:**
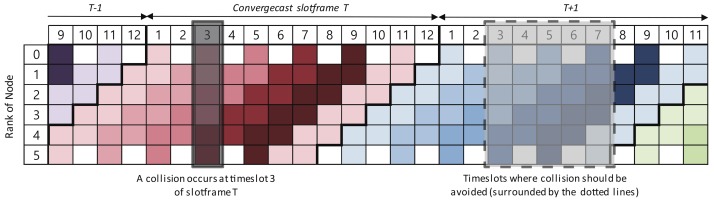
Timeslots of the convergecast slotframe where collisions should be avoided.

If a collision occurs at timeslot ts of the convergecast slotframe *T*, collisions should be avoided in the timeslots [$ts,ts+H_{max}\left(G\right)-1$] of the next slotframe T+1 so that the delayed packets could be transmitted to the sink without a collision. Figure 6 shows the timeslots of the convergecast slotframe where collisions should be avoided in network *G* with N=6. In Figure 6, the maximum hop counts of the packet forwarding path $H_{max}\left(G\right)$ are five. If the transmission of packets is delayed due to a collision with the baseline slotframe at timeslot ts=3 in convergecast slotframe *T*, collisions should be avoided in the timeslots from 3–7 of convergecast slotframe T+1 to transmit the delayed packets to the sink. If a collision occurs in the timeslots from 3–7 of slotframe T+1, the packets not transmitted in the slotframe *T* is delayed until the slotframe T+2.

The length of the slotframe and the number of channels should be relatively prime to allow each timeslot to communicate using all available channels [3,11]. In addition, $L(S_{Conv})$ and $L(S_{Base})$ should be satisfying the following three conditions, so that all nodes can send one packet to the sink within two cycles of the convergecast slotframe, even if a collision occurs. First, when the remainder obtained by dividing $L(S_{Conv})$ by $L(S_{Base})$ is defined as *m* in Equation (8), *m* should not be equal to zero; because if *m* is zero, a specific timeslot of $S_{Conv}$ at every cycle is suppressed by the shared timeslot of $S_{Base}$.

(8) $$m=(L\left(S_{Conv}\right)\;\mathrm{mod}\;L\left(S_{Base}\right))\neq 0.$$

As a second condition, if $L(S_{Base})$ is greater than $L(S_{Conv})$, $L(S_{Base})$ should be greater than or equal to $L\left(S_{Conv}\right)+H_{max}\left(G\right)$ as in Equation (9). When $L(S_{Base})$ is greater than or equal to $L\left(S_{Conv}\right)+H_{max}\left(G\right)$, if a collision occurs at timeslot ts of convergecast slotframe *T*, then the next collision occurs after $ts+H_{max}\left(G\right)-1$ timeslots of slotframe T+1. Therefore, a collision does not occur in the timeslot section [$ts,ts+H_{max}\left(G\right)-1$] of slotframe T+1, so delayed packets in slotframe *T* could be transmitted to the sink.

(9) $$L\left(S_{Base}\right)\geq \left(L\left(S_{Conv}\right)+H_{max}\left(G\right)\right),\;if\;L\left(S_{Base}\right)>L\left(S_{Conv}\right).$$

**Figure 7:**
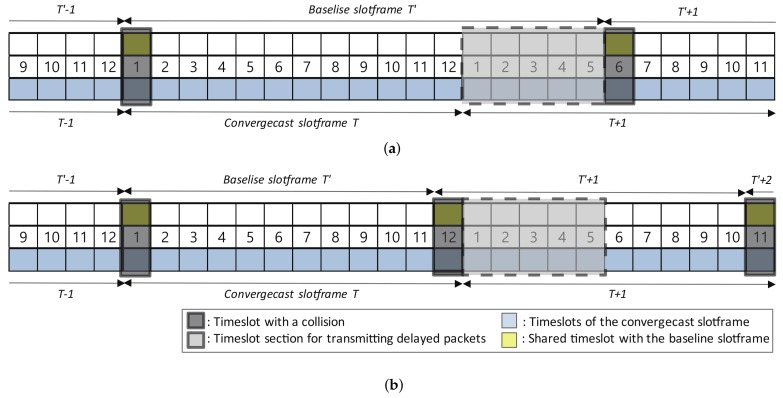
Timeslot section where collisions should be avoided. (**a**) A case where the baseline slotframe size (17) is greater than the convergecast slotframe size (12); (**b**) a case where the baseline slotframe size (11) is smaller than the convergecast slotframe size (12).

Figure 7a shows an example where $L(S_{Base})$ is greater than $L(S_{Conv})$. In the example, a collision occurs at timeslot ts=1 of slotframe *T* and timeslot ts=6 of slotframe T+1. Since collisions do not occur in timeslot Section 1, Section 2, Section 3, Section 4 and Section 5 of slotframe T+1, the packets delayed at timeslot ts=1 of slotframe *T* could be transmitted to the sink.

In the last condition, if $L(S_{Base})$ is less than $L(S_{Conv})$, then $L(S_{Base})$ should be greater than $H_{max}\left(G\right)+m-1$ as in Equation (10). If a collision occurs at ts timeslot of slotframe *T*, collision will occur at $ts+L(S_{Base})-m$ timeslot of slotframe T+1. Since no collision should occur at [$ts,ts+H_{max}\left(G\right)-1$] of slotframe T+1, $L(S_{Base})-m$ should be less than zero or greater than $H_{max}\left(G\right)-1$. *m* cannot be larger than $L(S_{Base})$ since *m* is the remainder obtained by dividing $L(S_{Conv})$ by $L(S_{Base})$ as in Equation (8). Therefore, $L(S_{Base})$ should be larger than $H_{max}\left(G\right)+m-1$.

(10) $$L\left(S_{Base}\right)>H_{max}\left(G\right)+m-1,\;if\;L\left(S_{Base}\right)<L\left(S_{Conv}\right).$$

Figure 7b shows an example where $L(S_{Base})$ is less than $L(S_{Base})$. In the example, collisions occur at timeslot ts=1,12 in slotframe *T* and timeslot ts=11 in slotframe T+1. Since there is no collision in the timeslot section [1, 5] of slotframe T+1, a packet delayed at timeslot ts=1 in slotframe *T* can be transmitted from slotframe T+1 to the sink.

## 5. Analysis of Escalator

This section analyzes the performance and limitations of Escalator. Firstly, we prove the conflict-freeness of the convergecast slotframe schedule using the wireless conflict model. Then, we evaluate the performance of Escalator through the analysis of the end-to-end delay and bandwidth.

### 5.1. Conflict Definition

Wireless communications of nodes using a single wireless interface may cause conflicts. Wireless conflicts are distinguished by primary (explicit) and secondary (implicit) conflicts [14,16,29]. A node with a single wireless interface cannot transmit and receive at the same time and cannot simultaneously receive from two or more nodes. We call a conflict caused by the limitation of the single wireless interface a primary conflict.

**Definition** **1.** *A primary conflict occurs in the following cases: 1. If a node simultaneously transmits and receives, 2. When a node simultaneously receives from two or more nodes.*

**Figure 8:**
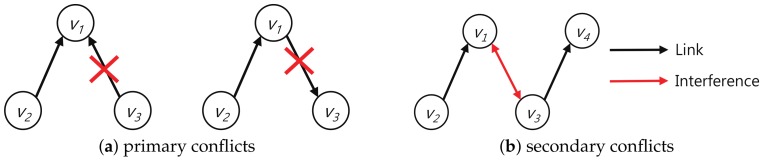
Conflicts in TDMA wireless networks.

Communications among nodes in a wireless environment have an interference area that can affect other communications. When some nodes are communicating, conflicts may occur if other nodes simultaneously communicate using the same channel in the interference area. A conflict resulting from interference between communications using the same channel and the same timeslot is referred to as secondary conflict. Figure 8a,b show primary and secondary conflicts, respectively.

**Definition** **2.** *A secondary conflict means a conflict that may occur among communications using the same channel and the same timeslot.*

### 5.2. Proof of the Conflict-Freeness of Convergecast Schedule

We prove that the schedule of the convergecast slotframe is conflict-free using the wireless conflict model. First, we prove that the schedule of the convergecast slotframe does not cause the primary conflict defined in Definition 1. The convergecast slotframe creates unicast and broadcast links between the parent and child nodes along the DODAG topology. We prove that the schedule generated by Escalator does not cause a primary conflict by showing that there is no conflict in the links between the parent and children.

**Theorem** **1.** *The unicast and broadcast links generated by the schedule of the convergecast slotframe do not cause the primary conflict.*

**Proof.**  We show the orthogonality of the unicast and broadcast links generated by the convergecast slotframe, proving that the primary conflict does not occur. A schedule for the convergecast slotframe of a node consists of the four operations assigned by the timeslot allocation mechanism, as described in Section 4.1. For node $v_{i}$, SET_BT(*i*) and SET_RX(*i*,*i*) assign $BT_{i}$ and $RX_{ii}$ operations to the same timeslot of i×2−1. However, since $v_{i}$ does not receive $v_{i}$ packets, $RX_{ii}$ is not used. SET_BR(*i*,*j*) and SET_TX(*i*,*j*) assign $BR_{ij}$ and $TX_{ij}$ operations to the same timeslot of j×2 for nodes $v_{i}$ and $v_{j}=p\left(v_{i}\right)$. However, since $v_{j}$ is the parent of $v_{i}$, $TX_{ij}$ is not used. In the convergecast slotframe, the broadcast links are always assigned to the timeslot for which unicast links are not used via SET_BT and SET_BR. Thus, unicast and broadcast links generated by the schedule of the convergecast slotframe are orthogonal. ☐

**Theorem** **2.** *There is no primary conflict among nodes with the same parent in the convergecast slotframe.*

**Proof.**  We show the orthogonality of the schedule among nodes with the same parent in the convergecast slotframe, proving that the primary conflict does not occur. Let nodes $v_{i}$ and $v_{j}$ (i≠j) have the same parent, then $SG\left(v_{i}\right)⋂SG\left(v_{j}\right)=\emptyset$. Therefore, the schedules of $v_{i}$ and $v_{j}$ assigned by the timeslot allocation mechanism do not use the same timeslot except BR operations. Furthermore, since $H_{v_{i}}=H_{v_{j}}$, the schedule of $v_{i}$ and $v_{j}$ is not influenced by the sliding slot frame. Therefore, the schedules of $v_{i}$ and $v_{j}$ are orthogonal to each other. ☐

**Theorem** **3.** *There is no primary conflict between the parent and child nodes in the convergecast slotframe.*

**Proof.**  We show that in the convergecast slotframe, it is impossible for the parent and the child nodes to transmit or receive data simultaneously, thus proving that the primary conflict does not occur. Let nodes $v_{i}$ and $v_{j}\in C\left(v_{i}\right)$. By the timeslot allocation mechanism, $v_{i}$ and $v_{j}$ assign TX and BR to even timeslots and assign RX and BT to odd timeslots. Since $H_{v_{j}}=H_{v_{i}}+1$, the schedule of $v_{j}$ is shifted one more left than the schedule of $v_{i}$ by the sliding slotframe mechanism. Therefore, if $v_{i}$ executes TX in even timeslots and executes RX in odd timeslots, $v_{j}$ executes TX in odd timeslots and executes RX in even timeslots. As with unicast, if $v_{i}$ executes BT in odd timeslots and executes BR in even timeslots, $v_{j}$ executes BT in even timeslots and executes BR in odd timeslots. Therefore, it is not possible to transmit or receive data simultaneously in one timeslot between two nodes that have a parent child relation. ☐

**Corollary** **1.** *The schedule of the convergecast slotframe does not cause the primary conflict.*

**Proof.**  The convergecast slotframe creates unicast and broadcast links only between the parent and child nodes along the DODAG topology. The unicast and broadcast links of the convergecast slotframe do not cause the primary conflict by Theorem 1. There is no primary conflict between parent and child nodes by Theorem 2 and Theorem 3. Therefore, the schedule of the convergecast slotframe does not cause the primary conflict. ☐

The secondary conflict may occur when two or more links are communicating simultaneously on the same channel, as defined in Definition 2. Escalator uses the channel offset selection mechanism to isolate channels between links using the same timeslot to prevent the secondary conflict. Theorem 4 proves that the convergecast slotframe does not generate the secondary conflict.

**Theorem** **4.** *There is no secondary conflicts between the links generated by the convergecast slotframe.*

**Proof.**  By the channel offset selection mechanism in Section 4.3, the communication channels used by the two links having a rank difference of two or more are different. Furthermore, by Theorem 2 and Theorem 3, the communication of two links whose rank difference is one or less does not execute in the same timeslot. Therefore, since the links using the same timeslot generated by the convergecast slotframe communicate using different channels, the secondary conflict does not occur. ☐

We prove that the schedule of the convergecast slotframe is conflict-free through Corollary 1 and Theorem 4.

### 5.3. Performance Analysis

#### 5.3.1. End-to-End Delay Analysis by the Overlap of Convergecast and Baseline Slotframes

We analyze the average end-to-end delay of packets transmitted from a node to the sink in Escalator. The convergecast slotframe provides a consecutive timeslot schedule where nodes can send packets to sinks with minimal delay. However, the packet transmission delay may increase due to a collision caused by slotframe overlap. We calculate the average end-to-end delay of the packet transmission from a node to the sink considering the delay due to the slotframe overlap. The $S_{Conv}$ and $S_{Base}$ combined schedule is repeated for each Least Common Multiple (LCM) timeslot of both $L(S_{Conv})$ and $L(S_{Conv})$. We define *r*, the number of times $S_{Conv}$ is repeated during $LCM(L\left(S_{Base}\right),L\left(S_{Conv}\right))$ timeslots, as in Equation (11).

(11) $$r=\frac{LCM(L\left(S_{Base}\right),L\left(S_{Conv}\right))}{L(S_{Conv})}.$$

The end-to-end delay of packet transmission from node $v_{i}$ to the sink using the convergecast slotframe is $H_{v_{i}}$ timeslots. If a slotframe collision occurs while a packet is being delivered to the sink, the end-to-end delay of this packet increases to $H_{v_{i}}+L\left(S_{Conv}\right)$ timeslots. Let *d* be the number of packets delayed by collision during $LCM(L\left(S_{Base}\right),L\left(S_{Conv}\right))$ timeslots. In Escalator, node $v_{i}$ can send one packet to the sink in two cycles of the convergecast slotframe, so *d* cannot exceed r/2. Each node transmits r−d packets to the sink during $LCM(L\left(S_{Base}\right),L\left(S_{Conv}\right))$ timeslots by schedule collision, and the collision-induced delay increase occurs in *d* packets. Therefore, we sum up the delays in r−d packet transmissions and use them to calculate the average end-to-end delay. Equation (12) represents the average end-to-end delay of node $v_{i}$.

(12) $$D_{e2e}\left(v_{i}\right)=\frac{\left(r-2d\right)\times H_{v_{i}}+d\times \left(H_{v_{i}}+L\left(S_{Conv}\right)\right)}{r-d}\;timeslots.$$

#### 5.3.2. Bandwidth and Buffering Capability

In Escalator, The bandwidth of the node is determined by the size of the convergecast slotframe. In a network with *N* nodes, the size of the convergecast slotframe $L(S_{Conv})$ should be greater than N×2. The node then transmits r−d packets to the sink while $S_{Conv}$ repeats *r* times. The bandwidth Escalator provides to each node in network *G* is computed as follows:

(13) $$B\left(G\right)=\frac{1}{L(S_{Conv})\times Dt}\times \frac{r-d}{r}\;packets/second,$$

where Dt is the duration of the timeslot. As *N* increases, $L(S_{Conv})$ increases, and bandwidth B(G) per node decreases. Conversely, when *N* decreases, B(G) increases.

We analyze the amount of buffer used to transmit convergecast traffic at each node. A node receives and transmits a packet using consecutive links. Thus, each node uses one buffer to transmit convergecast traffic. If a packet is delayed due to a collision, it will remain in the buffer until the next cycle of the convergecast slotframe. Each node has a delay of packet transmission due to collision of up to $\left\lceil \frac{L(S_{Conv})}{L(S_{Base})}\right\rceil$ times for each cycle of the convergecast slotframe. Therefore, the minimum buffer amount for each node of network *G* for the convergecast traffic transmission in Escalator is calculated as follows:

(14) $$Q\left(G\right)=1+\left\lceil \frac{L(S_{Conv})}{L(S_{Base})}\right\rceil packets.$$

## 6. Evaluation

We implement Escalator using Contiki [30], an open source platform for IoT. We verify the operation of the implemented Escalator using a simulator and hardware devices. We measure the performance factors of Escalator and existing autonomous scheduling schemes using a simulation and a testbed. After that, we evaluate the performance of Escalator using the measured performance factors.

### 6.1. Experimental Setup

We measure the performance of Escalator with 6TiSCH-minimal [24] and Orchestra [8]. 6TiSCH minimal uses a slotframe containing a shared timeslot and idle timeslots. All nodes of 6TiSCH-minimal perform contention-based communication using the shared timeslot. In the evaluation, 6TiSCH minimal is expressed as minimal, and the size of the slotframe is 5.

Orchestra uses three slotframes separated by the traffic type. The EB slotframe is used for the transmission of the beacon, and its size is 397. The broadcast slotframe is used for transmission of the RPL control messages, and its size is 31. The unicast slotframe is used for the transmission of application traffic and consists of a sender-based shared timeslot in the evaluation. Orchestra is expressed as OrchestraX, and *X* is the size of the unicast slotframe.

Escalator uses the baseline and convergecast slotframes. The size of the baseline slotframe is 31, equal to the size of the broadcast slotframe of Orchestra. The maximum hop counts of the network are 20. In evaluation, Escalator is expressed as EscalatorX, and *X* is the size of the convergecast slotframe.

**Figure 9:**
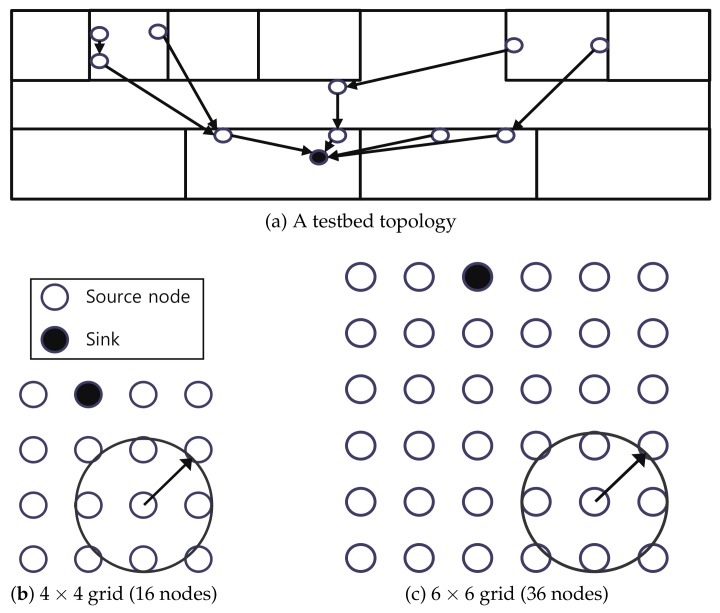
Testbed and simulation topologies.

We use a testbed and a simulation to measure the performance of Escalator and existing autonomous scheduling schemes. First, the testbed consists of 11 devices using the NXP JN5168 platform. The devices of the testbed are divided into a sink and 10 source nodes. Figure 9a shows the network topology used in the testbed. In the testbed, we use the following settings for each scheduling scheme. 6TiSCH-minimal uses a slotframe size of 5 (minimal). Orchestra uses a unicast slotframe size of 29 (Orchestra29). Escalator uses a convergecast slotframe size of 73 (Escalator73). We use the packet transmission interval of 20 s and 5 s for each scheduling scheme. In the experimental, all nodes send 2000 packets at the same interval up to the sink.

Second, we run the simulation using Cooja [31]. Cooja is a Contiki network simulator that provides hardware-level emulation based on Mspsim (Java-based instruction level simulator for the MSP430 microcontroller) [32]. In the simulation, the network topology uses 16 and 36 nodes arranged in a square grid as shown in Figure 9b,c. We run the simulation using the settings for each scheduling scheme described below. 6TiSCH-minimal uses a slotframe size of 5 (minimal). Orchestra uses two settings; the size of the unicast slotframe for each setting is 37 and 53, respectively (Orchestra37, Orchestra53). Escalator uses two settings; the size of the convergecast slotframe for each setting is 73 and 107, respectively (Escalator73, Escalator107). The simulation also uses the packet transmission interval of 20 s and 5 s as in the case of the testbed.

We use the following network settings for the testbed and the simulation. The channel hopping uses 16 channels. The timeslot duration of TSCH uses 20 ms so that the reception processing of the packet could be completed within one timeslot. The packet buffer size of the node in the testbed is 16. In the simulation, the packet buffer size of the node is 12. Each node can retransmit packets up to 8 times. The application traffic of the nodes uses UDP packets of a 24-byte payload.

### 6.2. Performance Metric

We use the following performance factors to measure the performance of Escalator and existing scheduling schemes.

- The packet delivery ratio (PDR) is the percentage of packets successfully delivered to the sink. The PDR is measured as the ratio of the number of packets received by the sink to the total number of packets transmitted by nodes.
- The end-to-end delay means the number of timeslots that a packet takes to reach from a source node to the sink. We measure the ASN when the packet leaves from the source node and the ASN when the packet arrives at the sink. The difference between these two ASNs is used to calculate the end-to-end delay. We evaluated the performance by measuring the average of the end-to-end delay by the number of hops of the packet.
- The duty cycle means the ratio of the radio usage time to the total operation time of nodes. The duty cycle is measured only in the simulation, and CPU usage time and radio transmit and receive times are measured while transmitting 2000 packets for each node. We evaluated the performance by measuring the duty cycle by the number of routes in the node.

### 6.3. Testbed Results

**Figure 10:**
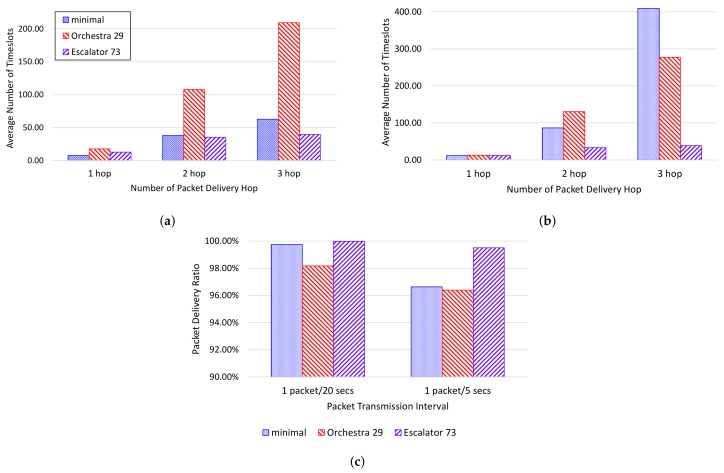
Testbed results: (**a**) end-to-end delay by packet transmission hop when the packet transmission interval is 20 s; (**b**) end-to-end delay by packet transmission hop when the packet transmission interval is 5 s; (**c**) PDR per the packet transmission interval.

Figure 10 represents the end-to-end delay and PDR for each scheduling scheme in the testbed. Figure 10a shows the end-to-end delay for each packet delivery hop counts in 20-s intervals. Experimental results show that Orchestra has the largest increase in end-to-end delay with increasing packet delivery hop counts. Orchestra can receive packets from multiple child nodes within a single slotframe, but only one packet can be sent to the parent. Thus, when a packet is transmitted across multiple hops, the end-to-end delay increases proportionally to the slotframe size. On the other hand, Escalator shows a smaller end-to-end delay than Orchestra because packet transmission from the source to sink is performed in one slotframe.

Figure 10b shows the end-to-end delay for each packet transmission hop counts in 5-s intervals. In the experimental results, 6TiSCH-minimal shows a higher end-to-end delay increase compared to the case of the 20-s interval. 6TiSCH-minimal performs contention-based communication unlike the other two scheduling schemes. Therefore, if collision increases due to node traffic increase, end-to-end delay increases by packet retransmission.

Figure 10c shows the PDR per packet transmission cycle. When the packet transmission interval is 20 s, all three scheduling schemes show a PDR of more than 98%. When the packet transmission interval is 5 s, the PDR of 6TiSCH-minimal and Orchestra is reduced to 96%. Due to the increase of retransmission and the lack of timeslots for packet transmission, the time for the packet to remain in the buffer of the node becomes longer and the packet is dropped due to the lack of the buffer of the node. However, Escalator shows 99% or more PDR regardless of the packet transmission interval due to the collision-free schedule and the use of fewer buffers by consecutive timeslots.

### 6.4. Simulation Results

**Figure 11:**
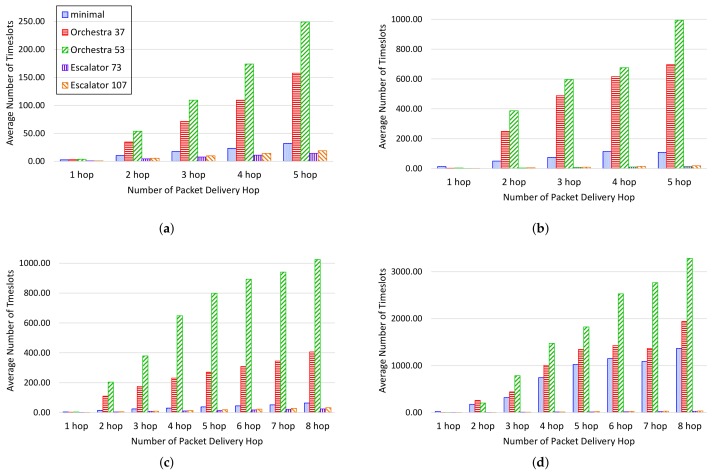
End-to-end delay per packet delivery hops in the simulation: (**a**) a packet transmission interval of 20 s on the 4×4 grid; (**b**) a packet transmission interval of 5 s on the 4×4 grid; (**c**) a packet transmission interval of 20 s on the 6×6 grid; (**d**) a packet transmission interval of 5 s on the 6×6 grid.

Figure 11 shows the end-to-end delay per packet delivery hop counts in packet transmission in the simulation. In the simulation, the maximum number of packet delivery hop counts is five hops in the 4×4 grid and eight hops in the 6×6 grid. Figure 11a,b shows the end-to-end delay when the interval of packet transmission is 20 s and 5 s on the 4×4 grid. 6TiSCH-minimal and Orchestra increase the end-to-end delay as node traffic increases. In contrast, the end-to-end delay of Escalator can be seen to remain constant with increasing traffic of the node.

Figure 11c,d shows the end-to-end delay when the interval of packet transmission is 20 s and 5 s in the 6×6 grid. In Figure 11c,d, we can see results similar to those of the testbed for the end-to-end delay. 6TiSCH-minimal shows that the end-to-end delay increases as the node traffic increases due to contention. Orchestra provides a conflict-free schedule, but does not provide enough timeslots for the number of child nodes and traffic growth of the node since only one packet per slotframe cycle can be sent to the parent. As a result, Orchestra shows an increase in the end-to-end delay as the number of network nodes and the traffic of nodes increase. Escalator shows that the end-to-end delay remains constant even as the number of nodes in the network increases and the traffic of the nodes increases.

**Figure 12:**
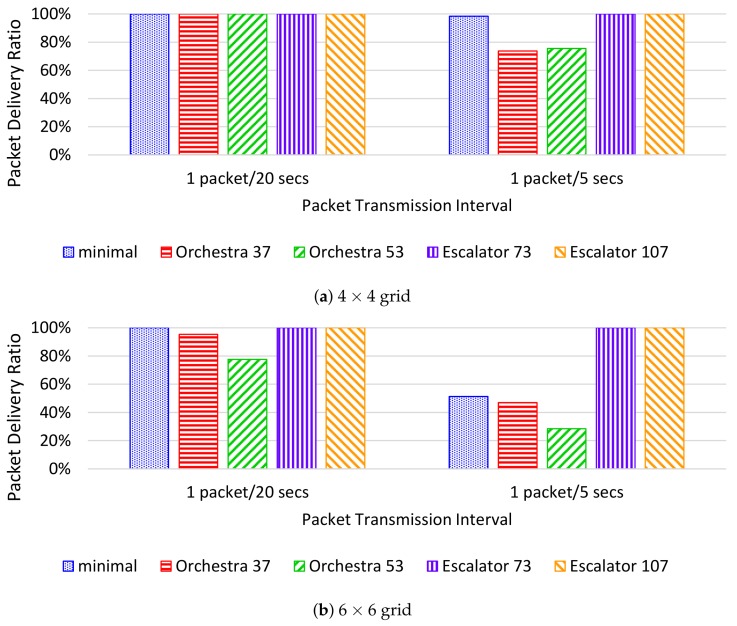
PDR per packet transmission interval in the simulation.

Figure 12a shows the PDR when the interval of packet transmission is 20 s and 5 s in the 4×4 grid. If the interval of packet transmission of the 4×4 grid is 20 s, the PDR of Orchestra37 and Orchestra53 is 100%. However, if the interval of packet transmission in the 4×4 grid is 5 s, the PDR of Orchestra37 and Orchestra53 is reduced to 74% and 76%, respectively. Orchestra cannot process the increased traffic of the nodes, resulting in packet loss due to the packets accumulating in the node’s buffer.

Figure 12b shows the PDR in the 6×6 grid. In this case, a packet drop occurs not only in Orchestra, but also in 6TiSCH-minimal. In contrast, Escalator shows a PDR of 100% even when the number of nodes in the network and the traffic of the nodes increase. Escalator can transmit application traffic within a limited end-to-end delay regardless of the number of transmitted packets within the available bandwidth.

Table 2 shows the average and maximum duty cycle of the nodes measured in the simulation. 6TiSCH-minimal shows an average duty cycle of 2.4% under all simulation conditions. This is because all the nodes in 6TiSCH-minimal use the same schedule of the shared communication method regardless of the network condition and the traffic requirement. The average duty cycle of Orchestra increased from 0.89%–1.31% depending on the number of nodes and packet transmission period. The average duty cycle of Escalator increased from 0.78%–1.54%. Although Escalator shows better performance in the packet delivery ratio and the end-to-end delay than Orchestra, the average duty cycle does not increase significantly.

**Figure 13:**
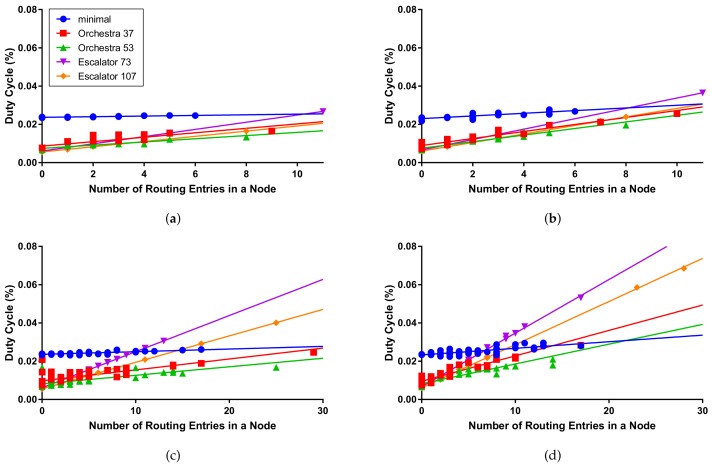
Duty cycle per number of routing entries in a node: (**a**) a packet transmission interval of 20 s on the 4×4 grid; (**b**) a packet transmission interval of 5 s on the 4×4 grid; (**c**) a packet transmission interval of 20 s on the 6×6 grid; (**d**) a packet transmission interval of 5 s on the 6×6 grid.

The maximum duty cycle of Escalator in the simulation increases to 6.85%. We can see in Figure 13 that the duty cycle increase for the number of routing entries in the Escalator node is greater than that of Orchestra. In Escalator, the amount of scheduled timeslot increases in proportion to the number of routing entries. Therefore, the number of routing entries increases with the node closer to the sink, which increases the use of the radio to transmit packets received from the child nodes to the sink.

## 7. Conclusions

In this paper, we propose a new autonomous scheduling scheme for the convergecast. Escalator provides a slotframe schedule for a network in which all nodes can send packets to the sink in every slotframe cycle, regardless of the network topology. Escalator minimizes the end-to-end delay from a source node to the sink by creating a schedule that considers multichannel and packet forwarding order. Escalator schedules using only the DODAG topology and node IDs and does not generate traffic overhead used for network information collection and scheduling negotiation between nodes. We prove the conflict-freeness of the schedule generated by Escalator using the wireless conflict model. Furthermore, we analyze the average end-to-end delay of packet transmission and the available bandwidth for each node in the network.

We implement Escalator based on Contiki, which is widely used as the OS platform of IoT. The performance is evaluated by comparing with the existing autonomous scheduling schemes using a testbed and simulation. As a result, it is confirmed that Escalator maintains the delay of the packet transmission at a constant level irrespective of the increase of the number of nodes and the increase of the traffic. In addition, Escalator shows lower end-to-end delay and a higher packet delivery ratio for the convergecast compared to existing autonomous scheduling schemes.

Experimental results show that Escalator does not significantly increase the average duty cycle, even for better performance in the PDR and the end-to-end delay compared to the existing autonomous scheduling schemes. In addition, we can see that the duty cycle increase for the number of routing entries of the node is higher in Escalator than in the other scheduling schemes. This phenomenon means that the energy consumption of the nodes around the sink is increased, and as a result, the network lifetime can be reduced. We will study the routing considering the balance of the remaining energy and routing topology for the increase of the network lifetime [33,34,35]. In addition, Escalator provides a conflict-free schedule for convergecast traffic only. We consider not only the monitoring, but also the reliability improvement through the conflict-free schedule for the control plane. To this end, we will continue to work on the creation of schedules for downward traffic that can be used with Escalator.

## Figures and Tables

**Table 1:** Main notations.

Symbol	Definition
*G*	The network consisting of nodes *V* and links *E*
*N*	The number of nodes in the network
*V*	The set of nodes in the network
*E*	The set of links in the network
DT	Duration of timeslot
Ch	The number of available orthogonal channel
CO	The channel offset that is used for multiple channel usage at a single timeslot
$CO_{Op}$	The channel offset used for Op
ASN	The total number of timeslots that have elapsed since the start of the network
$S_{Conv}$	The convergecast slotframe
$S_{Base}$	The baseline slotframe
L(S)	The size of slotframe *S* in timeslot unit
$v_{i}$	A node with ID *i*
$p(v_{i})$	The preferred parent of node $v_{i}$
$C(v_{i})$	The set of the direct child nodes of node $v_{i}$
$SG(v_{i})$	The set of nodes in the sub-graph of node $v_{i}$, excluding $v_{i}$ itself
$l_{ij}$	A link for which the sender is $v_{i}$ and the receiver is $v_{j}$
$H_{v_{i}}$	Hop count of node $v_{i}$, the number of hops from the sink to node $v_{i}$
Op(S,ts)	Operation at timeslot ts in slotframe *S*, which is one of the following four operations
$BT_{i}$	The operation in which $v_{i}$ broadcasts a beacon
$BR_{ij}$	The operation in which $v_{i}$ receives a beacon sent by $v_{j}$
$TX_{ij}$	The operation in which $v_{i}$ transmits a unicast packet originated from $v_{j}$
	to the parent node $p(v_{i})$
$RX_{ij}$	The operation in which $v_{i}$ receives a unicast packet, originated from $v_{j}$,
	from one of the child nodes $C(v_{i})$
$\left[ts_{1},ts_{2}\right]$	The timeslot section from $ts_{1}$ to $ts_{2}$
LCM(a,b)	Least common multiple of *a* and *b*
$H_{max}\left(G\right)$	Maximum hop counts of the packet-forwarding path in network *G*
$D_{e2e}\left(v_{i}\right)$	Average end-to-end delay of node $v_{i}$
B(G)	Average bandwidth of node in network *G*
Q(G)	Average required buffer capability of nodes in network *G*

**Table 2:** Measured average and max duty cycle in the simulations.

	Duty Cycle (%)							
	4×4 grid, 20 s		4×4 grid, 5 s		6×6 grid, 20 s		6×6 grid, 5 s	
	Average	Max	Average	Max	Average	Max	Average	Max
minimal	2.40	2.47	2.44	2.77	2.41	2.61	2.48	2.96
Orchestra 37	1.06	1.65	1.20	2.56	1.15	2.46	1.31	2.83
Orchestra 53	0.89	1.34	1.02	1.96	0.96	1.68	1.08	2.10
Escalator 73	0.92	2.67	1.12	3.65	1.20	3.05	1.54	5.31
Escalator 107	0.78	1.64	0.98	2.39	0.99	4.01	1.32	6.85
